# Evaluation of New Antimicrobial Materials Incorporating Ethyl Lauroyl Arginate or Silver into Different Matrices, and Their Safety in Use as Potential Packaging

**DOI:** 10.3390/polym13030355

**Published:** 2021-01-22

**Authors:** Sofía Manso, Magdalena Wrona, Jesús Salafranca, Cristina Nerín, María José Alfonso, Miguel Ángel Caballero

**Affiliations:** 1 Department of Analytical Chemistry, Aragón Institute of Engineering Research I3A, EINA-University of Zaragoza, Torres Quevedo Building, María de Luna 3, 50018 Zaragoza, Spain; sofia.mnsn@gmail.com (S.M.); magdalenka.wrona@gmail.com (M.W.); fjsl@unizar.es (J.S.); 2 Nurel, R+D Department, Polígono Malpica, Ctra. Barcelona km. 329, 50016 Zaragoza, Spain; mjalfonso@samca.com (M.J.A.); acaballero@samca.com (M.Á.C.)

**Keywords:** antimicrobial packaging materials, ethyl lauroyl arginate, silver, biopolymer, migration, PS, LDPE

## Abstract

A big challenge for today’s industry is antimicrobial preservation and the safety of food. An effective solution to this problem can be a modern invention such as antimicrobial packaging. In the presented research the antimicrobial activity of two new active films incorporating silver, as IONPURE IPL, and ethyl lauroyl arginate (LAE) were evaluated, by employing a low-density polyethylene (LDPE) matrix and a biofilm material, respectively. Additionally, LAE was also incorporated into polystyrene (PS) pads by two different methods: by spraying and by immersion of the PS pads into an aqueous LAE solution. LDPE films containing silver did not show any antimicrobial activity against *Escherichia coli* and *Aspergillus flavus,* whereas the biofilm containing LAE reduced the growth of *Salmonella enterica* but did not inhibit *Aspergillus flavus*. The active PS pads, both sprayed and immersed in LAE solution, also showed antimicrobial activity, causing a reduction of 99.99% of *Pseudomonas putida* growth. Thermal treatment at 180 °C for 6 and 15 min did not modify the antimicrobial activity of LAE against *Salmonella enterica.* Moreover, inductively coupled plasma-mass spectrometry (ICP-MS) analysis was performed to check the migration of silver from developed material intended for food packaging applications into food simulant.

## 1. Introduction

Food quality spoilage can be produced by physical, chemical, and biological factors. Microbial contamination is considered as the most dangerous, causing foodborne illnesses that affect the health of consumers. Moreover, microbial growth accelerates undesired changes in the aroma, color, and texture of food resulting in its shelf-life reduction. Therefore, antimicrobial preservation and safety of food are challenges for today’s food industries [1,2].

Among the emerging technologies, antimicrobial packaging is currently a promising approach to actively control foodborne pathogens [3,4]. Antimicrobial food packaging is based on novel materials that extend the lag phase and therefore inhibit the growth of microorganisms [2].

Antimicrobial packaging performance can be achieved either by indirect contact between food and packaging, in the case of volatile active agents, or by direct contact between the material and the food surface when considering non-volatile substances. In this latter case, the antimicrobial compound can be both immobilized on the material surface or directly incorporated into the polymer matrix to, subsequently, be released to the food. Therefore, antimicrobial packaging is particularly effective in food products where bacteriological contamination occurs [5].

The literature provides evidence of successful developments and applications of antimicrobial packaging mainly based on essential oils [6,7,8,9,10,11,12,13,14], but surprisingly there are very few effective antimicrobial packaging available on the market [15,16]. This is because the development and production of materials incorporating high antimicrobial activity compounds able to be effective in vivo are very challenging. Moreover, the incorporation of essential oils as antimicrobial agents often modifies the physicochemical properties of packaging and/or food, such as color or smell, making them unacceptable for consumers [17]. However, the application of antimicrobials such as ethyl lauroyl arginate (LAE) or silver avoids these inconveniences. LAE is an extremely active antimicrobial surfactant authorized as a food preservative by European Food Safety Authority (EFSA), Food and Drug Administration (FDA), and Food Standards Australia New Zealand (FSANZ) [18,19,20] with demonstrated applications as an active agent in food packaging [21,22,23,24]. Likewise, silver displays strong antimicrobial activity against different pathogens having low toxicity to human health [25].

The latest developed food packaging materials containing LAE were based on a chitosan-polyvinyl alcohol blend matrix [18]. The authors showed that very high concentrations of LAE (5 and 10%) were the most effective against common food bacterial pathogens including *Escherichia coli*. Further, polypropylene (PP) packaging coated with LAE showed an antimicrobial effect against *E. coli* at high concentration (10%) [26]. Concerning the use of silver as antimicrobial, several packaging materials were developed, but in all cases, silver was in form of nanoparticles (0.50% nanofibers [27] and 0.12–0.42% dextran-coated silver nanoparticles [28]). Significant differences between blank and antimicrobial films were obtained for *E. coli*, among others. This type of packaging is very challenging, as the nanoparticles should be stable and any migration of them has to be demonstrated.

The present paper aims to design, develop, and compare new antimicrobial materials intended for packaging applications, containing LAE or silver as active substances. To the best of our knowledge, LAE has been incorporated and its antimicrobial activity evaluated for the first time into two packaging materials: polystyrene (PS) pads, and a biofilm matrix (INZEA F19).

**Figure 1:**
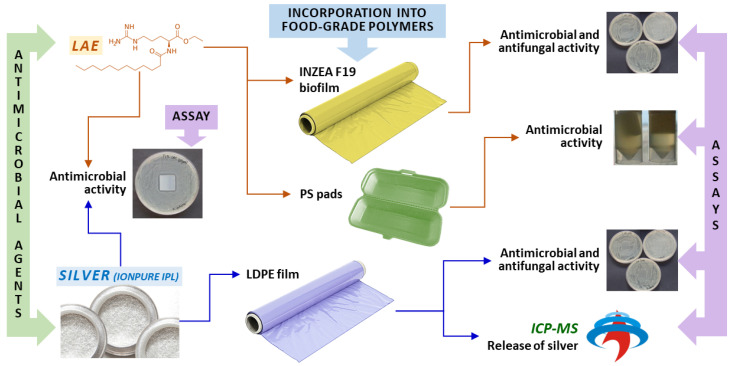
Flowchart of the different experiments carried out during the present study (colors of the polymers do not correspond to the actual ones).

On the other hand, limited research has been reported about active packaging based on IONPURE IPL, a silver-containing glass antimicrobial powder which releases silver ions gradually in the presence of water or moisture. According to the manufacturer [29], IONPURE IPL is safe (acute oral toxicity in mice >2000 mg/kg, no skin irritation was observed in albino rabbits, no skin sensitization was reported on guinea pigs, and mutagenicity tests were negative). Moreover, it shows high antimicrobial performance at low concentration, maintains the optical properties of polymers, and minimally modifies the mechanical properties of the material, such as tensile strength, bending strength or impact strength. In the present study, the performance of low-density polyethylene (LDPE) films prepared by incorporating IONPURE IPL has been explored. In addition to their antimicrobial activity assessment, migration tests into different food simulants have been performed and the dissolved silver determination has been carried out by inductively coupled plasma-mass spectrometry (ICP-MS). Figure 1 summarizes the flowchart of the different experiments carried out. The obtained results are shown and discussed.

## 2. Materials and Methods

### 2.1. Chemicals

Acetic acid (≥99.7%, CAS 64-19-7) was purchased from Sigma-Aldrich, Madrid, Spain, ethanol (≥99.9%, CAS 64-17-5) was from Scharlab, Barcelona, Spain. Ethyl lauroyl arginate (CAS 60372-77-2) was supplied by VEDEQSA S.A., Barcelona, Spain. Inorganic silver antimicrobial IONPURE IPL powder (1.8% silver by weight, from silver–magnesium–calcium–phosphate–borate complex [30]) was supplied by Ishizuka Glass Co., LTD, Iwakura, Japan. ICP multi-element standard solution IV (23 elements, 1000 mg/L each, in diluted HNO_3_, including Ag) was from Supelco, Madrid, Spain.

LDPE and INZEA F19 biofilm were from NUREL Company, Zaragoza, Spain. The extruded PS foam was from Bandesur Alcalá S.A., Jaén, Spain.

Ultrapure water was produced by a Wasserlab Ultramatic GR system, Barbatáin, Spain.

### 2.2. Bacterial and Mold Strains

*Escherichia coli* ATCC 25922 (*E. coli*) and *Salmonella enterica* CECT 556 (*S. enterica*) were chosen to evaluate the antimicrobial activity of films with both silver and LAE, whereas *Pseudomonas putida* CECT 324T (*P. putida*) was used to assess the antimicrobial activity of PS pads with LAE. The mold *Aspergillus flavus* CECT 2687 *(A. flavus)* was selected to study the antifungal activity of films with both silver and LAE. Mueller-Hinton Broth (MHB), Plate Count Agar (PCA), Tryptic Soy Agar (TSA), and Potato Dextrose Agar (PDA) were purchased from Scharlab, Barcelona, Spain.

### 2.3. Antimicrobial Films Containing Ag and LAE

Antimicrobial agents were incorporated into LDPE film and INZEA F19 biofilm by the company NUREL, part of SAMCA group. Both materials were prepared by extrusion from masterbatches with antimicrobial compounds. INZEA F19, suitable as a material for food contact applications, is a transparent, flexible, bio-based (85% renewable content), biodegradable, and compostable (according to EN 13432) polylactic acid (PLA) grade suitable for cast film extrusion, with stiffness similar to PP. The recommended processing temperatures are: feed section: <25 °C, barrel zones: 140–160/170/175–180/175–185 °C, adapter: 175–185 °C, die: 180–185 °C. Melting temperature is 175–185 °C, and >210 °C should be avoided. All the processes are protected by Patent EP1923423B1.

The following films were prepared:

- BK—Blank LDPE
- Ag_20—LDPE film with incorporated 2% of IONPURE IPL (0.036% silver), 20 µm thickness
- Ag_50—LDPE film with incorporated 2% of IONPURE IPL (0.036% silver), 50 µm thickness
- BK—Blank INZEA F19 biofilm
- LAE_20—INZEA F19 biofilm with incorporated 6% of LAE, 20 µm thickness
- LAE_50—INZEA F19 biofilm with incorporated 6% of LAE, 50 µm thickness

### 2.4. LAE Polystyrene (PS) Foam Pads Preparation

**Figure 2:**
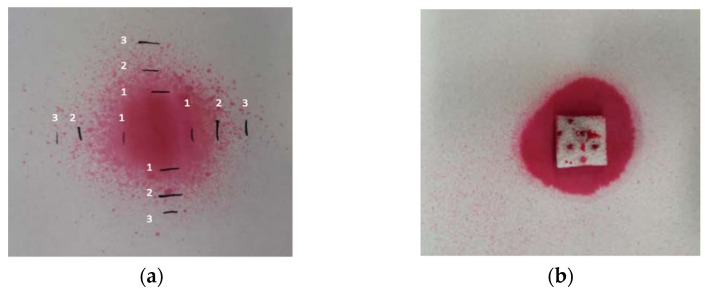
Distribution of an aqueous solution of Allura red AC observed when performing (**a**) 1 spray with different heights such as 2 cm (line 1), 3.3 cm (line 2), and 4.5 cm (line 3); (**b**) 3 sprays at previously optimized fixed height (4.7 cm).

The objective of this experiment was to optimize the application of LAE on PS pads by spraying and by total immersion to a subsequent on-line treatment in packaging plants. Since intrinsically the final deposited concentration is greatly reduced in both methods, high initial concentrations of LAE (30 mg/mL for spraying and 22 mg/L for total immersion) were prepared. To maximize the precision, both LAE solution and ultrapure water as control were prepared in identical glass spray bottles. The PS pads were cut into pieces of approximately 2 cm × 2 cm (0.5 cm thickness) and weighed before spray application. To evaluate the antimicrobial activity of two LAE concentrations, half of the PS pieces were sprayed once and the other half twice without allowing the material to dry. The nozzle of the spray bottle was set always in the middle of the PS piece. To get the maximum reproducibility, the height and the angle of the spray were optically optimized (Figure 2) by using an aqueous solution of Allura red AC to ensure the same LAE distribution on the pad surface. Five replicates were prepared for each type of piece. All pieces were left to dry at room temperature for 24 h, then they were weighed to determine the amount of dry LAE incorporated in the pores of the PS pad surface. Later, LAE would release and dissociate in a liquid medium to perform its antimicrobial activity.

In the case of total immersion, the PS pads were horizontally oriented and completely submerged for 6 s in the 22 mg/mL aqueous LAE solution. Then, the pads were left to dry at room temperature, and weight was daily controlled until ensuring the total evaporation of water.

### 2.5. Antimicrobial Activity of Silver-Containing Powder

In this experiment, the antimicrobial activity of IONPURE IPL powder, containing silver, was evaluated. Following the EFSA opinion [31], IONPURE IPL was selected since it is authorized to food contact applications. An initial concentration of 32 mg/mL was prepared by dissolving the powder in ultrapure water. Afterward, the stock solution was firstly diluted by a factor of 1:10, from which serial dilutions (1:2) in microtubes were made. All the dilutions were performed in duplicate, being the silver final concentration in the range from 0.006 to 3.2 mg/mL. In all cases, MHB was used as culture broth. Finally, 50 μL of an *S. enterica* inoculum were added to all the microtubes, being the final tested concentration 10^5^ CFU/mL. All the samples were then incubated for 24 h at 37 °C under constant shaking, after which the minimum inhibitory concentration (MIC)—the lowest concentration that prevented visible growth in a liquid medium—was determined. Subsequently, the minimum bactericidal concentration (MBC)—the lowest concentration that killed at least 99.9% of the initial bacterial population—was obtained after sowing 20 µL aliquots of each of the microtubes in PCA culture medium, incubating the Petri dishes for another 24 h at 37 °C.

### 2.6. Antimicrobial and Antifungal Activity of Silver Films

To evaluate antimicrobial activity, both control and active Petri dishes were inoculated in PCA culture medium, with 100 μL of a suspension of 10^6^ CFU/mL of *E. coli*. The pieces of films (2 cm × 2 cm) were placed in direct contact with the culture medium, and Petri dishes were incubated for 24 h at 37 °C. For each type of film three replicates were prepared.

Similarly, to study the antifungal activity, all the Petri dishes were inoculated in a PDA culture medium with 100 µL of a suspension of 10^6^ CFU/mL of *A. flavus*, placing the 2 cm × 2 cm pieces of films in direct contact with the culture medium, and incubated for 7 days at 25 °C. In this case, two replicates were performed for each type of film.

### 2.7. Assessment of Silver Release from the Active Films by ICP-MS

The silver release as a consequence of migration tests was carried out by total immersion of films in three different aqueous food simulants (95% ethanol, 10% ethanol, and 3% acetic acid) using the area-to-volume ratio established in EU Regulation 10/2011 (6 dm^2^/1 kg simulant). The experiment was carried out in triplicate at 60 °C for 10 days. Blank of simulant was also prepared in each case. After the migration test, the simulants were analyzed by inductively coupled plasma-mass spectrometry (ICP-MS, Agilent 7500a, Palo Alto, CA, USA) to determine the migrated silver. The two stable isotopes ^107^Ag and ^109^Ag were measured for higher accuracy by checking the expected isotopic ratio (51.84% ^107^Ag/48.16% ^109^Ag). Samples of 10% ethanol and 3% acetic acid were analyzed directly, while samples of 95% ethanol were diluted 10 times to avoid plasma extinction when organic content is higher than 10%. The isotope detection was carried out in full-spectrum mode, with 0.3 s per mass and 5 repetitions. Peristaltic pump aspiration speed was set at 0.3 revolutions per second.

Two series of silver standard solutions in the range of 1–100 µg/L were prepared to obtain external calibration curves, the first one in 10% (*v*/*v*) ethanol and the second one in 3% (*w*/*v*) acetic acid.

Analytical parameters of the method such as limits of detection (LOD) and quantification (LOQ) and linearity of calibration curves were determined.

### 2.8. Antimicrobial Activity and Thermal Stability of LAE

The antimicrobial activity of LAE was evaluated after subjecting both pure powder and 80 mg/mL aqueous solution of LAE to 180 °C for 6 and 15 min, respectively. LAE without heat treatment was chosen as reference. When considering powder, the same amount of LAE was accurately weighed in all replicates and placed in empty bottles previously sterilized.

The oven was preheated at 180 °C and empty bottles were tempered. After the heat treatment, the bottles were left to cool down to avoid breaking the glass. Then LAE was dissolved in sterilized ultrapure water. The samples were left for about 1 h on an orbital shaker at 37 °C, to favor their complete dissolving.

The 80 mg/mL stock solution was initially diluted by a factor of 1/100, from which serial dilutions (1/2) were made with MHB. Thus, the approximate final concentrations in the microtubes under analysis were: 800, 400, 200, 100, 50, 25, and 12.5 µg/mL, all of them performed in duplicate. Then, the methods of MIC and MBC evaluation described in Section 2.5 were applied.

### 2.9. Antimicrobial and Antifungal Activity of LAE Films

For this study, 100 μL of a suspension of 10^6^ CFU/mL of *S. enterica* were inoculated into PCA culture medium contained in both control and active Petri dishes. All films with LAE were evaluated by placing 2 cm × 2 cm pieces in direct contact with the culture medium. For each type of film, three replicates were considered. All Petri dishes were incubated for 24 h at 37 °C.

To perform the antifungal experiment, 100 µL of a suspension of 10^6^ CFU/mL of *A. flavus* was inoculated in PDA Petri dishes, both control, and active samples. Once again, the films (2 cm × 2 cm) were placed in direct contact with the culture medium in duplicate and incubated for 5 days at 25 °C

### 2.10. Antimicrobial Activity of LAE PS Pads

The pieces of active PS pads prepared both by spraying and total immersion were evaluated by submersion in Falcon tubes containing 9 mL of MHB, with 10^6^ CFU/mL of a suspension of *P. putida*. After assuring that the pads were continuously in contact with the culture broth, the tubes were left to incubate on an orbital shaker at 0.65 g (30 °C, 24 h). In addition to the control sample, a second control tube with the inoculated broth but without the pad was considered.

After the incubation, 20 µL aliquots were taken from each tube and diluted in sterile physiological serum. Then, 50 µL aliquots were sown in TSA Petri dishes and incubated for 24 h at 30 °C. Finally, the degree of antimicrobial activity was measured by the obtained colony count.

### 2.11. Statistics

Unless stated otherwise, all the samples were prepared in triplicate. The obtained results are presented as the average value ± standard deviation. A significance level expressed as *p*-value, of 0.05 has been selected from a statistical point of view.

## 3. Results and Discussion

### 3.1. Antimicrobial Activity of Silver Powder

The IONPURE IPL powder showed low antimicrobial activity against *S. enterica*, requiring relatively high concentrations to inhibit growth in a liquid medium (MIC 0.8–1.6 mg/mL) and to reduce microbial growth in the Petri dishes (MBC 1.6–3.2 mg/mL). Similar results were reported in other researches involving silver nanoparticles against *Staphylococcus aureus* (*S. aureus*) [32], with MIC and MBC values of 0.625 mg/mL, or IONPURE IPL against *Streptococcus mutans* (*S. mutans*) [33], finding an MIC of 7.5 mg/mL. Such high concentrations can be explained by the fact that the antibacterial powders can be gradually deposited at the bottom of the dishes due to its limited solubility, which cannot be completely avoided even when shaking. Consequently, the sedimentation prevents that all the available amount of antimicrobial agent could react with the bacteria. This was confirmed when investigating six inorganic antimicrobial agents, including IONPURE H, [34] against *S. mutans* and *S. aureus*, among others. The MBC results in agar (1.25 and 5 mg/L, respectively) were higher than those in TSA (0.625 and 2.5 mg/L, respectively).

### 3.2. Antimicrobial and Antifungal Activity of Silver Films

The LDPE films with incorporated silver as IONPURE IPL did not show antimicrobial activity in any case, either against *E. coli* or against *A. flavus*. These findings contrast with those given by the manufacturer [29], stating 99.9% reduction in the population of both *E. coli* and *S. aureus* when exposed to PP plates containing 0.1% IONPURE IPL. Nevertheless, our results are coherent with other studies reporting little or none antimicrobial activity of IONPURE IPL incorporated in different polymers. An investigation on the effect of resin plates containing IONPURE IPL against *S. mutans* [33] noticed MIC of 5%, higher than the concentration initially present (2%) in the formulations of active films developed in the present paper. Other studies [35,36] compared the antimicrobial performance of LDPE films containing triclosan and IONPURE against *S. aureus* and *E. coli*. Whilst triclosan films reduced the bacteria population up to of 99.9%, low antimicrobial efficiency was obtained in both untreated LDPE films and those containing IONPURE, detecting large amounts of bacteria adhered on the film surfaces. The explanation could be related to the availability of dissolved silver, as will be discussed in the Section 3.3.

### 3.3. Release of Silver from Active Films by ICP-MS

The following analytical parameters were obtained: linearity range for all calibration curves was 0.08–100 μg/L; LOD and LOQ for both silver isotopes in two simulants (3% acetic acid and 10% ethanol) were 0.02 and 0.08 μg/L, respectively. The correlation coefficient (r^2^) for both silver isotopes in 3% acetic acid was 0.9998 and in 10% ethanol was 0.9996.

Table 1 presents the results of the silver release, measured as ^107^Ag and ^109^Ag, from different films to food simulants after migration tests. The highest migration value was obtained in active films immersed in 3% acetic acid. On the other hand, the lowest one was obtained for active films immersed in 95% ethanol, which was foreseeable due to the influence of both acidity [37] and polarity [38] of the solvents, as described in previous studies.

These values are fully consistent with the absence of antimicrobial activity shown by the LDPE films commented on Section 3.2. Martinez-Abad et al. [39] measured the silver released from different PLA films containing silver nitrate, and antimicrobial effect against *S. enterica* was evident at concentrations higher than 18 µg/L, with substantial variations in the range 10–20 µg/L, and without any inhibitory effect below 6 µg/L.

In the case of acetic acid, significant differences (*p*-values were 0.025 and 0.029 for ^107^Ag and ^109^Ag, respectively) were found when studying the effect of the active film thickness, as could be expected. Higher migration occurred in the case of the thicker film due to its higher absolute content of silver. In contrast, when comparing active films in contact with 10% ethanol, no significant differences, with p-values of 0.213 (^107^Ag) and 0.247 (^109^Ag), were observed, probably due to the low affinity of silver for the solvent. This fact was confirmed when increasing the ethanol percentage up to 95%, where no migration was detected at all. Even in the worst case (50 µm film in contact with 3% acetic acid), the immobilization of silver into the polymeric matrix was very effective, since less than one-thousandth of the total content was transferred to the liquid simulant, in agreement with previous studies with similar materials and test conditions [40,41].

Regarding the safety in the use of the developed films, the Council of Europe has suggested a specific release limit (SRL) of 80 µg/kg food for silver [42]. Therefore, considering the density of simulants as 1 g/mL, none of the active films exceed the SRL. Consequently, they can be used in direct food-contact applications, providing that silver nanoparticles are not present.

### 3.4. Thermal Stability and Antimicrobial Activity of LAE

**Figure 3:**
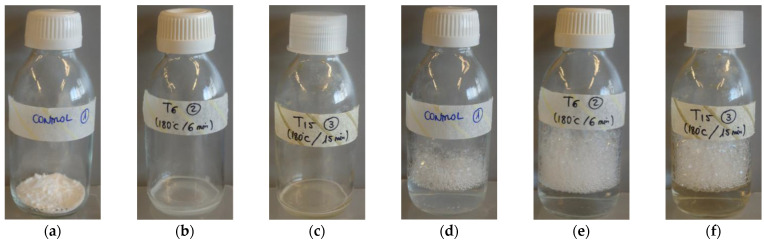
(**a**) Ethyl lauroyl arginate (LAE) without thermal treatment (control); (**b**) LAE heated at 180 °C for 6 min (T6)*; (**c**) LAE heated at 180 °C for 15 min (T15)*; (**d**) unheated LAE dissolved in ultrapure water (control); (**e**) LAE heated at 180 °C for 6 min dissolved in ultrapure water (T6); (**f**) LAE heated at 180 °C for 15 min dissolved in ultrapure water (T15). * Samples just removed from the oven (melting temperature about 58 °C).

The heating of samples for 15 min at 180 °C slightly modified the visual appearance of the aqueous solutions of LAE, whereas those treated at 180 °C for 6 min had the same appearance as the unheated control samples, as shown in Figure 3. Nevertheless, changes became more evident during the dissolution of LAE samples subjected to heat treatment in ultrapure water, requiring longer solubilization times in all the cases.

The lowest tested concentration of LAE (11.5 µg/mL) produced identical results to the blank and caused no effect on the growth of the bacteria. The MIC resulted to be always the same (23 µg/mL); that is, in both the heated and unheated samples. Finally, the MBC was 46 µg/mL in all cases, which corresponded to the total absence of growth in the entire Petri dish. Consequently, very high antimicrobial activity was observed against *E. coli* and *S. enterica*. Despite it has been reported that LAE can decompose at temperatures higher than 107 °C [43], or in aqueous solutions [22], no significant differences were observed (*p*-value > 0.05) among samples, thus indicating a high stability of LAE in short-term heating. This fact can be considered very positive, since no loss of antimicrobial performance is expected in, for instance, heat sterilization or hot-filling processes.

### 3.5. Antimicrobial and Antifungal Activity of LAE Films

**Figure 4:**
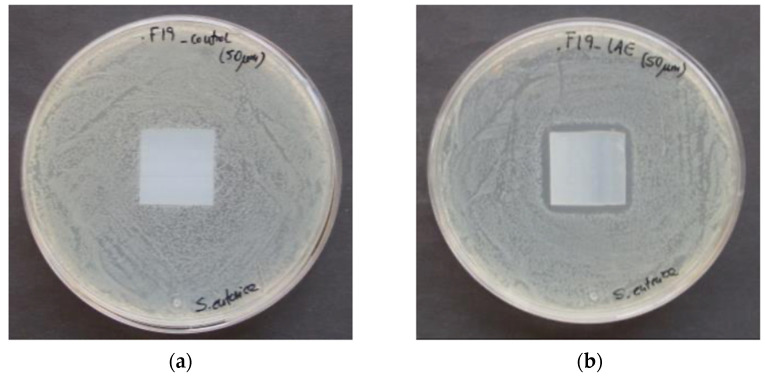
Petri dish with control film and 50 μm LAE film used against *S. enterica* after 24 h of incubation at 37 °C where (**a**) control sample; (**b**) 50 µm biofilm with LAE.

Despite the good results obtained by direct contact with LAE, the antimicrobial activity against *S. enterica* of biofilms with LAE was limited, since the inhibition halo around the 20 µm active film was 0.89 ± 0.33 mm (average considering the three replicates of the sample, and the measurements from both directions), whereas the one corresponding to the 50 μm LAE biofilm was 2.19 ± 0.58 mm. Furthermore, both LAE films failed to reduce the growth of *A. flavus*. To explain these results, one of the reasons is that the mobility and actuation mechanism of LAE [44] is greatly reduced due to its effective linking into the biofilm. The second reason is the different sensitivity between *S. enterica* and *A. flavus*. Hence, in previous works, the MIC of LAE against another strain of *A. flavus* was 400 µg/mL [45], whereas in the case of *S. enterica* was 25 µg/mL [17]. Figure 4 shows the growth of *S. enterica* after 24 h of incubation, with control film and 50 µm biofilm fortified with 6% LAE.

### 3.6. Amount of LAE Present in PS Pads

When considering sprayed PS pads, the amount of dry LAE after 1 and 2 spraying cycles was 0.91 ± 0.32 and 1.38 ± 0.16 mg/cm^2^, respectively. In the case of total immersion, the amount of dry LAE on the PS pad was 1.95 ± 0.22 mg/cm^2^ (considering only one side). Significant differences among treatments (*p*-value < 0.05) were found in all the cases. Total immersion was demonstrated to be the most effective, but it must be pointed out that the effectively treated area was one side (4 cm^2^) for spraying, and two sides plus four lateral surfaces (12 cm^2^) for total immersion. Taking into account the area normalization, no significant differences were observed (*p*-value = 0.064 for one spray vs. total immersion, and 0.073 for two sprays vs. total immersion).

### 3.7. Antimicrobial Activity of LAE PS Pads

**Figure 5:**
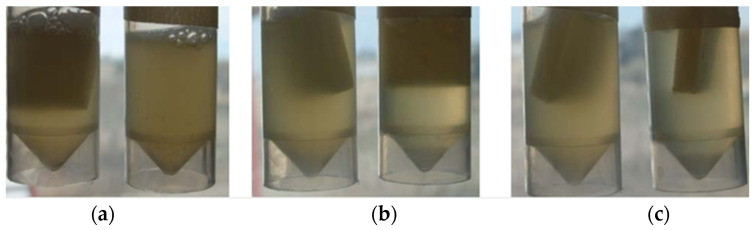
Macroscopic appearance of the tubes with *P. putida* after 24 h incubation at 30 °C, where (**a**) control sample of the pad with water (on the left) and without pad (on the right); (**b**) control sample of the pad (on the left) and pad with 1 spray of LAE (on the right); (**c**) control sample of the pad (on the left) and pad with 2 sprays of LAE (on the right).

The results of the antimicrobial evaluation in liquid medium of PS pads with LAE obtained by spraying can be seen in Figure 5. It can be observed that the culture broth showed evident turbidity after 24 h of incubation, which is an indicator of increased bacterial growth. This circumstance occurred in both the control without a pad and the tubes with pads (with and without ultrapure water). However, in all tubes containing the active pad with LAE, a reduction in turbidity occurred, thus indicating a decrease in bacterial growth.

The reduction of macroscopic growth shown in Figure 5 was confirmed with the aliquots extracted from each tube on TSA Petri dishes. Thus, while the growth of (6.75 ± 2.12) × 10^8^ CFU/mL was obtained for control pads, the active ones with LAE showed a 99.99% reduction (more than three logarithms) compared to the initial bacterial inoculum in all the cases, with no significant differences. The antimicrobial effect was achieved with both 1 and 2 spraying cycles per PS pad, as well as with those prepared by total immersion, producing total inhibition of *P. putida* in vitro after 24 h. In comparison to biofilms (Section 3.5) pads demonstrated to be much more effective, because LAE removal is considerably easier from PS surface. Therefore, these pads could be used in future assays during in vivo evaluation. In addition, these promising results will allow its straightforward implementation at an industrial level, mainly in spraying lines, where LAE concentration can be easily adjusted to fulfill the flow requirements of nozzles.

## 4. Conclusions

In contrast to the information provided by the manufacturer, none of the active LDPE films containing silver as IONPURE IPL exhibit antimicrobial activity either against *E. coli* or *A. flavus*, Nevertheless, similar results were already reported, being the most likely reason the concentration of released silver to the selected food simulants (8 µg/L or lower in our case vs. 18 µg/L required for full inhibition). As proposal for future work, the addition of higher amount of IONPURE IPL to the polymer formulation and/or the selection of other materials, such as PP, polyamide or PLA will be considered in order to increase the migration of silver, thus improving the antimicrobial effect.

LAE demonstrated to be thermally stable, without significant antimicrobial differences between heated and unheated samples. This behavior makes possible the use of LAE in food applications where high temperatures are reached, such as heat sterilization. Active materials containing LAE showed different results. In the case of INZEA F19 biopolymer, average antimicrobial activity was observed due to the efficient LAE linkage to the matrix, with a limited release of LAE against *S. enterica*. No inhibitory effect against *A. flavus*, due to its different sensitivity, was observed at all. The film thickness had no practical effect on the results. Regarding PS pads, homogenous deposition of LAE was noticed by both spraying and immersion without significant difference among methods. Total inhibition of *P. putida* was observed in all cases due to the higher availability of LAE. Consequently, active PS pads containing LAE could serve as a reference to be tested at pilot plant scale before its transfer to industrial level, with minimal changes in existing packaging lines.

## Figures and Tables

**Table 1:** Results of the silver release (measured as ^107^Ag and ^109^Ag) from different films into three food simulants by inductively coupled plasma-mass spectrometry (ICP-MS). Values are mean ± standard deviation (in µg/L).

	3% Acetic Acid		10% Ethanol		95% Ethanol	
	^107^Ag	^109^Ag	^107^Ag	^109^Ag	^107^Ag	^109^Ag
Blank	<LOD	<LOD	<LOD	<LOD	<LOD	<LOD
Ag_20—LDPE	6.74 ± 0.65	6.69 ± 0.64	2.85 ± 0.68	3.04 ± 0.65	<LOD	<LOD
Ag_50—LDPE	8.13 ± 0.57	8.05 ± 0.63	2.46 ± 0.06	2.50 ± 0.07	<LOD	<LOD

## Data Availability

The data presented in this study are available on request from the corresponding author.
